# American Medical Association

**Published:** 1874-06-15

**Authors:** 


### AMERICAN MEDICAL ASSOCIATION.

THE twenty-seventh annual meeting of this national organization was held in Detroit, commencing on the morning of June 2d, 1874. The members assembled in the Music Hall, and were called to order at 11 o'clock A. M., by the President, Dr. J. M. Toner, of Washington, and prayer was offered by Rev. Bishop McCroskey, of Michigan.

The hall was well filled with members, and the gallery with ladies and citizens. Dr. W. Brodie of Detroit, Chairman of the Committee of Arrangements, in a short, but appropriate speech, welcomed the members of the Association to the hospitalities of the citizens of Detroit, and congratulated them on the continued prosperity of the Association.

After reading the list of names so far as registered, and calling the roll of the Society, the President, Dr. J. M. Toner, delivered his annual address, which was listened to with great interest, both by the members and citizens.

The list of special committees was called, and such reports as were announced ready, were referred to the appropriate sections for consideration. Several volunteer papers were also announced and referred.

This completed the work of the first morning session.

At 2½ o'clock P. M., the several sections were called to order by their respective officers, and proceeded directly to the reading and consideration of the reports and papers that had been referred to them. In the Section on Practical Medicine, Materia Medica, and Physiology, Dr. Bulkley read an interesting paper on the nature and treatment of Eczema, which elicited some remarks from Drs. Grover, Woodward, McLaughlin, Pierce, Gray, Octerloony, Johnson and Canfield, after which it was referred to the Committee of Publication. Dr. P. J. Farnsworth, of Iowa, read a paper on the "Therapeutic Uses of Ammonia," which, after a brief discussion, was referred back to its author, with permission to publish it in such medical periodical as he might choose.

In the Section on Obstetrics and Diseases of Women, Dr. T. Parvin presented some instruments for inspection, and Dr. Beck, of Fort Wayne, Indiana, read a paper on the Theory of Generation. It was followed by a discussion participated in by Drs. J. P. White, of Buffalo; W. H. Byford, of Chicago; J. M. Sims, of New York; and M. A. Pallen, of St. Louis.

The Section on Surgery and Anatomy was presided over by Dr. S. D. Gross, of Philadelphia. Dr. A. Dunlap, of Springfield, Ohio, read a paper on "Enchondroma over the Sternum," which was referred to the Committee of Publication. Dr. E. M. Moore, of Rochester, New York, read an interesting paper on "Epiphyseal Fracture of the Superior Extremity of the Humerus," which elicited remarks from Drs. Quimby, of New Jersey; Hughes, of Iowa; Keller, of Kentucky; Reyburn, of Washington; Gross, of Pennsylvania; and Gregory, of Missouri; after which the paper was referred to the Committee of Publication.

Dr. Lewis A. Sayre, of New York, presented a report on Fractures, stating verbally an abstract of its contents. This led to an animated discussion by Dr. Sayre and Dr. Gregory, of St. Louis, pending which the Section adjourned.

The Section on Medical Jurisprudence and Chemistry met and adjourned without transacting any business.

The Section on State Medicine and Public Hygiene was called to order by its Chairman, Dr. A. N. Bell, of Brooklyn, New York. A paper by Dr. H. I. Bowditch, of Boston, concerning the establishment of State Boards of Health and of a National Sanitary Bureau, was read by the Secretary. Drs. R. C. Kedzie, of Michigan; A. B. Stewart, of Minnesota; and A. N. Bell, of Brooklyn, New York, also read abstracts of papers on the same subjects. Dr. Kedzie offered a resolution that the Association petition Congress for the establishment of a National Sanitary Bureau, which was discussed at considerable length by Drs. Kedzie, Brown, Cochrane, Bell, Westmoreland, Johnson, Thomas, Pratt, Van Demen, Waterhouse, Jones, Baker, Howard, Hitchcock and Grey; after which the resolution was adopted, and the Section adjourned.

On the morning of the second day the general meeting of the Association commenced at 9 o'clock, A. M., in the Opera House, the Music Hall having been found too small to accommodate the audience. After some preliminary business, the report of the Judicial Council in relation to the revision of the Code of Ethics, was read by Dr. N. S. Davis, Chairman of the Sub-Committee to whom the matter was referred last year. The report was unanimously adopted by the Association, and we will give it a place in the EXAMINER at an early day. The constitution was so amended as to exclude all medical institutions and organizations from the privilege of sending delegates, except State and Territorial medical societies; and such district, county, and city medical societies as are recognized by representation in their respective State societies; and the medical staffs of the army and navy,

Dr. J. M. Keller, of Louisville, Chairman of the Committee on the Relative Rank of the Medical Staff of the Army, reported progress, and the Committee was continued.

Dr. N. S. Davis, of Chicago, delivered his annual address as President of the Section on Practical Medicine, Materia Medica and Physiology. It occupied less than the prescribed limit of forty minutes; was well received, and referred for further consideration to the Section just named.

Dr. S. D. Gross, of Philadelphia, President of the Section on Surgery and Anatomy, delivered his annual address, which, although occupying a full hour and a half, was listened to with fixed attention to its close, when it was referred to the Section over which he presided, and the Association adjourned.

At 2½ P. M. the sections reassembled in their respective rooms, but the numbers in attendance were less, on account of a steamboat excursion on the river for the benefit of the ladies. In the Section on Practical Medicine, Materia Medica and Physiology, a paper on the "Indigenous Medical Botany of West Virginia," by Dr. E. A. Hildreth, was presented, but the author not being present, it was referred for examination to a subcommittee, consisting of the President of the Section, with directions to report to the Committee of Publication.

The report of Dr. Samuel J. Deal, on the Cultivation of the Cinchona Tree, was read, its recommendations adopted, and the committee continued. A paper of some length on the "Mechanism of the Encephalic Circulation," by Dr. R. A. Vance, was read by Dr. W. M. Carpenter, the author not being present. The paper elicited a brief discussion, after which it was referred back to the author, for publication in such medical journal as he might choose.

Dr. F. R. Buckham, of Flint, Michigan, read a paper on "Uremia and its Relations to Renal Disease," which elicited remarks from Drs. Bennett, Hyatt, Osterloony and Farnsworth, after which it was referred to the Committee of Publication. The Section then adjourned.

In the Section on Obstetrics and Diseases of Women, a paper was read on the "Inverted Uterus," and referred to the Committee of Publication. A new form of Pessary was presented, and its use explained by Dr. Scott, of Woodstock, Canada, which elicited an interesting discussion, participated in by Drs. Warner, of Boston; Miner, of Buffalo; Pallen, of St. Louis; White, of Buffalo; Byford, of Chicago; Ditherly, of Syracuse; and Morris, of Baltimore. A vote of thanks was tendered to Dr. Scott, and the Section adjourned.

In the Section on Surgery and Anatomy, the time of the session was wholly occupied with a continuance of the discussion on the Treatment of Fractures, elicited by the report of Dr. Sayre, presented yesterday. It was participated in by Drs. Garcelon, of Maine; Waterhouse, of Wisconsin; Sayre, of New York; Reed, of Ohio; Whiting, of Wisconsin; Quimby, of New Jersey; Hughes, of Iowa; Pierce, of Illinois; Gregory and Guillford, of Missouri; and King, of Pennsylvania. The report of Dr. Sayre was referred to the Committee of Publication, and the Section adjourned.

In the Section on State Medicine and Public Hygiene, Dr. R. C. Kedzie read a paper on the "Influence of Drainage on the Public Health." Papers on the same subject were also presented by Drs. Cabell, of Virginia, and Bell, of New York. A report by Dr. Robert D. Murray, Surgeon of U. S. M. H. Service, on the "Climatic Influences of Key West, was also presented. After some discussion, these several papers were referred to the Committee of Publication.

The Association met in general session at 9 o'clock A. M., of Thursday, a full attendance being present. After some items of miscellaneous business, Dr. T. Parvin, President of the Section on Obstetrics and Diseases of Women, read his address on Uterine Hemorrhage and Transfusion, which was listened to with great interest, and referred for consideration to the Section just named.

Dr. A. N. Bell, of Brooklyn, Chairman of the Section on State Medicine and Public Hygiene, read his address on the "Waste of Life." A vote of thanks was tendered to Dr. Bell, and his address referred to the Section for consideration. Reports from the Committee on Prize Essays, the Committee on Necrology, and the Librarian were received, and referred to the Committee of Publication. After some further miscellaneous business the Association adjourned.

The several Sections again assembled punctually at 2½ P. M., and were called to order by their respective officers. In the Section on Practical Medicine, Materia Medica and Physiology, Dr. L. D. Bulkley read a short paper on a New Anti-Pruritic Remedy, which was referred to the Committee of Publication. The special remedy alluded to was a combination of hydrate of chlor. and gum camphor, equal parts one drachm, and rose ointment one ounce, thoroughly mixed, and applied frequently to the surface affected with pruritis. The address of the President of the Section, Dr. N. S. Davis, was then taken up for consideration, and after remarks by Drs. McLaughlin, Quimby, Rouse, Chubbuck, Grover and Bulkley, it was referred to the Committee of Publication, and two special committees appointed to carry into practical effect some of its recommendations. Dr. Garrish, of New York, read a paper on the "History and Treatment of Hydrophobia," which, containing no new matter in relation to that disease, was referred back to the author for publication in the medical periodicals. Dr. E. W. Grey, of Illinois, read a paper on the "Relations of Physiology to Practical Medicine," which was briefly discussed and referred to the Committee of Publication. Dr. E. Seguin, of New York, briefly explained his method of Mathematical Thermometry. The last paper presented to this Section was on "Electricity as a Restorative Agent in Narcosis and Asphyxia," by Dr. Caldwell, of Baltimore. The paper was read by the Secretary, and referred back to its author, with the recommendation that it be published in the medical periodicals. The Section then adjourned *sine die*.

In the Section on Obstetrics and Diseases of Women, the subjects of Transfusion and Ovariotomy were discussed by Drs. Parvin, White, Forbes, Busey, Sims and Dunlap. The address of Dr. Parvin, as President of the Section, was referred to the Committee of Publication, and the work of the Section was completed for this year.

In the Section on Surgery and Anatomy, Dr. George M. Beard, of New York, presented a communication on the "Uses of Electricity in Surgery," giving the results of his experience in the application of electricity to goitre, malignant tumors, and some forms of cutaneous disease. The communication was referred to the Committee of Publication. The Section spent an hour in the examination of instruments and cases, and then adjourned *sine die*.

In the Section on Medical Jurisprudence and Chemistry, Dr. E. Lloyd Howard read an interesting paper on the "Legal Relations of Emotional Insanity," and Dr. A. N. Talley, one on the "Relations of Psychology to Medicine," both of which were referred to the Committee of Publication, and the work of that Section was closed.

In the Section on State Medicine and Public Hygiene, the time was all occupied in the consideration of miscellaneous matters and the appointment of committees for future work.

The fourth and last general session of the Association commenced at 9 o'clock A. M., of Friday. Two hours were occupied in hearing the reports of the Committee on Nominations; of the Committee of Publication; of the Treasurer; and in passing complimentary resolutions. The following officers were elected for the ensuing year:

*President*—W. K. Bowling of Tennessee.

*Vice Presidents*—Wm. Brodie, of Michigan; J. J. Woodward, of U. S. Army; H. W. Brown, of Texas; H. D. Didama, of New York.

*Section on Practical Medicine, Materia Medica and Physiology*—Dr. Austin Flint, of New York, Chairman; J. K. Bartlett, of Wisconsin, Secretary.

*Section on Obstetrics and Diseases of Women*—Dr. W. H. Byford, of Chicago, Chairman; S. C. Busey, of Washington, Secretary.

*Section on Surgery and Anatomy*—Dr. E. M. Moore, of Rochester, Chairman; T. S. Latimer, of Baltimore, Secretary.

*Section on Medical Jurisprudence and Chemistry*--Dr. J. Cochrane, of Mobile, Chairman; G. A. Moses, of St. Louis, Secretary.

*Section on State Medicine and Public Hygiene*—Dr. H. J. Bowditch, of Boston, Chairman; H. M. Baker, of Michigan, Secretary.

The place selected for the next annual meeting is Louisville, Kentucky.

We have given the foregoing simple outline of the doings of the recent meeting of the American Medical Association, that our readers may form some idea of the actual amount and kind of work done during the four days spent in Detroit. About 450 members were in attendance, including a large delegation from the Dominion of Canada. Dr. Toner presided with dignity and ability. The most perfect good order and harmony prevailed throughout, and we seriously doubt whether an equal number of men, belonging to any profession, ever met and accomplished more actual work in the same length of time, and with so much decorum and good feeling. The profession and citizens of Detroit were lavish in their hospitality, and the evening entertainments were delightful.

